# Cartilage Biopsy for Autologous Cell–Based Repair of the Knee in the Wide-Awake Setting Using Needle Arthroscopy

**DOI:** 10.1016/j.eats.2023.07.022

**Published:** 2023-10-23

**Authors:** Ian Savage-Elliott, Matthew T. Kingery, Mohammad T. Azam, Dylan T. Lowe, Eric J. Strauss

**Affiliations:** Department of Orthopedic Surgery, NYU Langone Health, New York, New York, U.S.A.

## Abstract

Chondral and osteochondral lesions of the knee are a commonly occurring pathology that can pose challenges to the treating surgeon. For the appropriate cartilage injury, autologous cell–based articular cartilage repair techniques have shown promising results. However, these treatments traditionally require 2 separate surgical procedures. Recent advances in needle arthroscopy technology have made it possible to conduct the first stage of autologous chondrocyte implantation surgery in the wide-awake office setting, mitigating cost and resource utilization. The purpose of this technical note is to serve as a proof of concept and describe the process of obtaining a cartilage sample in the wide-awake patient using a needle arthroscope.

Chondral injuries of the knee are a commonly occurring pathology present in upwards of 10% to 12% of the population.^1^ Given the poor capacity for articular cartilage to heal itself and the lack of gold-standard treatment options, these lesions present a challenging problem for the orthopaedic surgeon.^2^ Recently, there has been growing interest in the use of cell-based articular cartilage restoration procedures, which have shown promising results in treating full-thickness cartilage defects.^3^ Matrix-induced autologous chondrocyte implantation (MACI) is a 2-stage procedure that first involves arthroscopically harvesting cartilage from a non–weight-bearing surface of the injured knee. The harvested chondrocytes are then expanded in culture ex vivo and ultimately implanted into the defect during the second stage of the procedure.^2^^,^^3^ Despite promising results, there is some criticism of MACI owing to its 2-stage nature, which adds cost and utilization of hospital resources, as well as additional exposure to anesthesia.^4^^,^^5^ However, with the recent advances in needle arthroscopy technology, these disadvantages may be mitigated.

Improving on prior design, a recently developed needle arthroscopy system uses a 1.9-mm semi-flexible camera, which provides a minimally invasive option for diagnostic and therapeutic procedures in the office or operating room setting. The needle scope makes use of 0° chip-on-tip camera technology, which allows for manual bending of the scope up to 15° and produces an image quality comparable to that of traditional arthroscopy. Additionally, the needle scope system includes a variety of arthroscopic instruments, such as 2.0-mm shavers, burrs, small probes, graspers, and curettes, to facilitate minimally invasive procedures.

The purpose of this technical note is to serve as a proof of concept and describe chondrocyte harvest in the wide-awake setting. We describe considerations for obtaining adequate local anesthesia, proper indications, visualization tips, and the advantages of performing this procedure in a wide-awake setting. Additionally, we provide a step-by-step guide to performing the technique that, in combination with Video 1, will allow orthopaedic surgeons to reproduce the technique and make cell-based repair therapies more cost- and resource-effective.

## Surgical Technique

### Preoperative Planning and Positioning

**Fig 1:**
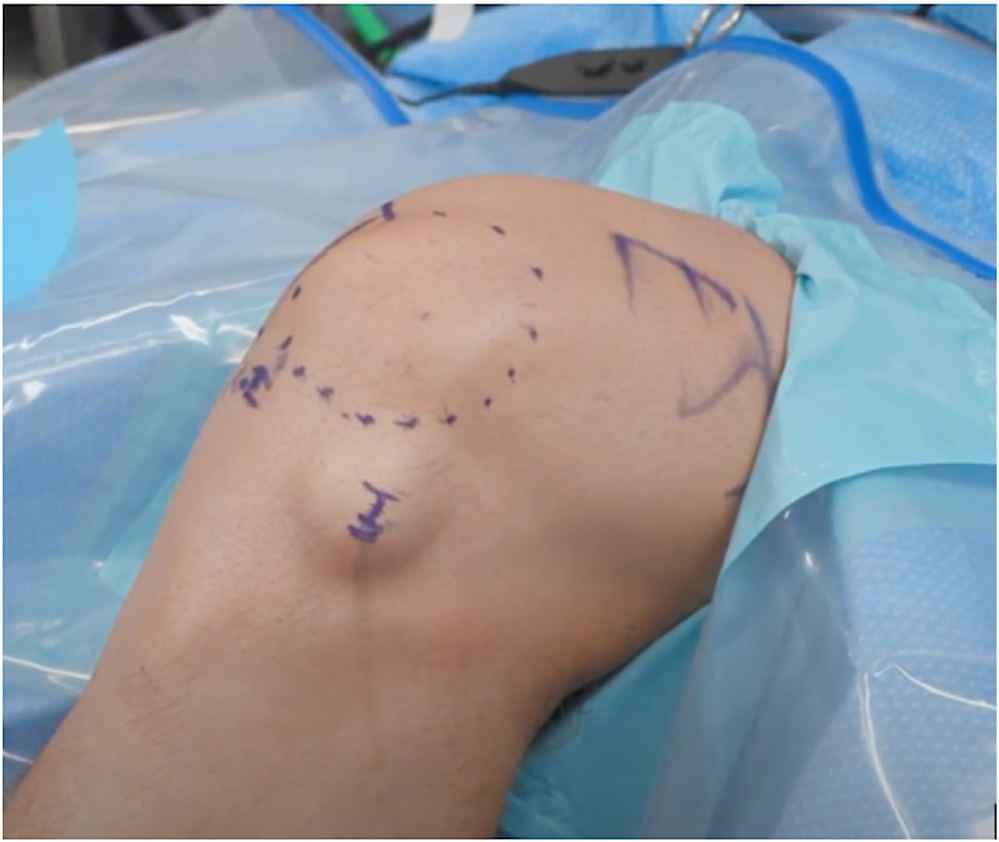
Standard knee setup for needle arthroscopy. A left knee is shown, flexed to 90°. The patient is positioned comfortably on the table, and the operative knee is prepared and draped in the usual sterile fashion. Local anesthetic has been infiltrated into the anterolateral portal in the left knee.

The surgical technique is described in detail in Video 1. The patient is positioned comfortably on the table in the supine position. If the patient prefers to watch the arthroscopic video as the procedure takes place, a monitor can be placed in the patient’s line of sight. A tourniquet is not placed because this would cause unnecessary discomfort to the wide-awake patient. Prior to the introduction of the needle arthroscope, the anterolateral and anteromedial portals are each injected with 10 mL of a 1:1 mixture of 1% lidocaine with epinephrine and 0.5% of bupivacaine. After a few minutes and verification with the patient that the local anesthetic has taken effect, another injection is performed through the anterolateral portal to ensure proper anesthesia, consisting of 20 mL of a 1:1 mixture of 1% lidocaine and 0.5% bupivacaine. The extremity is then prepared and draped in the usual sterile fashion (Fig 1). Anatomic landmarks and standard anterolateral and anteromedial portal sites are marked on the skin.

### Operative Technique

**Fig 2:**
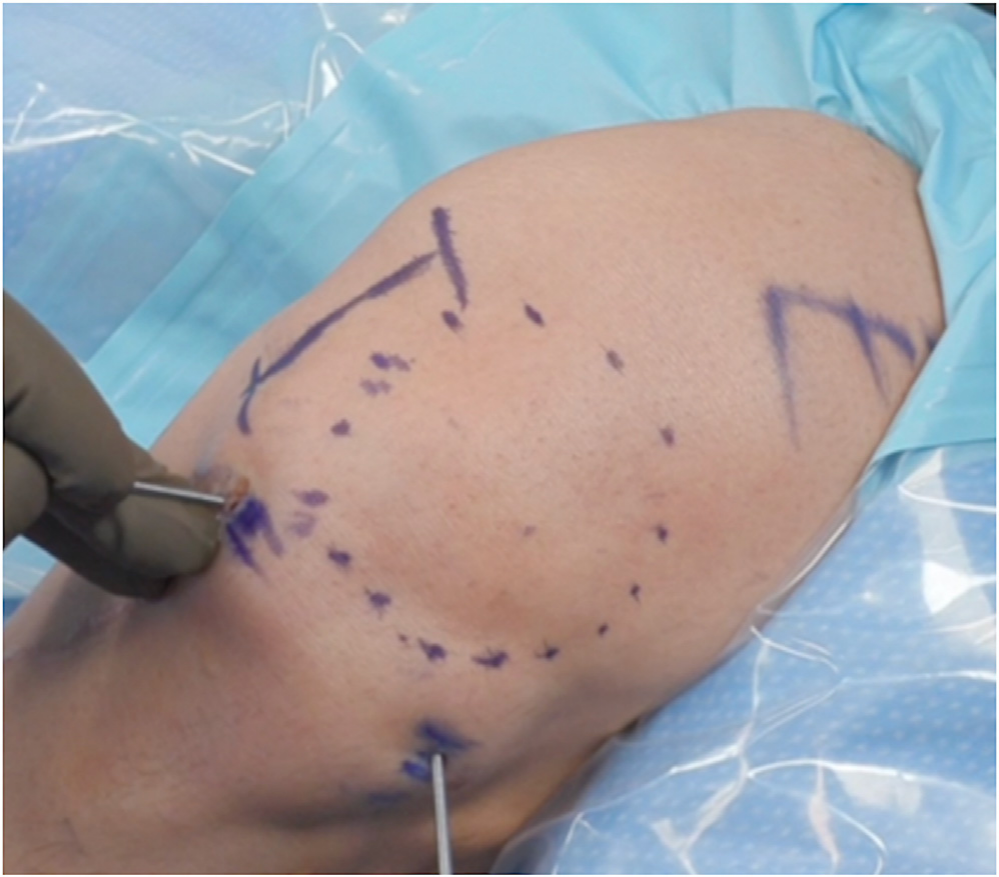
Needle arthroscopy approach in left knee, flexed to 90°, using standard anterolateral portal (viewing portal, right) and anteromedial portal (working portal, left).

A No. 11 scalpel is used to make a 2-mm stab incision at the anterolateral portal site (Fig 2). No undercutting of the capsule or spreading using a blunt clamp should be performed so that discomfort to the patient is minimized. Furthermore, this prevents extravasation of fluid through the portal around the needle arthroscope.

With the knee in full extension, a blunt trocar is used to enter the joint under the patella into the suprapatellar pouch in standard fashion. The camera is exchanged over the trocar and connected to the integrated inflow and outflow fluid management system (Dual-Wave; Arthrex, Naples, FL) at a pressure of 35 mm Hg. Fluid consists of 1 L of 0.9% normal saline solution mixed with 5 mL of epinephrine, which allows for adequate fluid flow and hemostasis for optimal visualization. The increase in intracapsular pressure associated with the initial introduction of fluid into the joint may be slightly uncomfortable for the patient. Therefore, the operating surgeon should maintain communication with the awake patient throughout the procedure. A diagnostic arthroscopy is then performed in the usual fashion.

Next, under direct visualization, the anteromedial portal is established. An 18-gauge spinal needle is introduced into the joint, and the portal site is confirmed. By use of a No. 11 scalpel, a 2-mm stab incision is made, again without undercutting of the capsule or blunt spreading. The spinal needle can then be exchanged for a probe to test the integrity of the relevant structures. A 2-mm shaver or grasper can be used to removed excess soft tissue to improve visualization as necessary.

**Fig 3:**
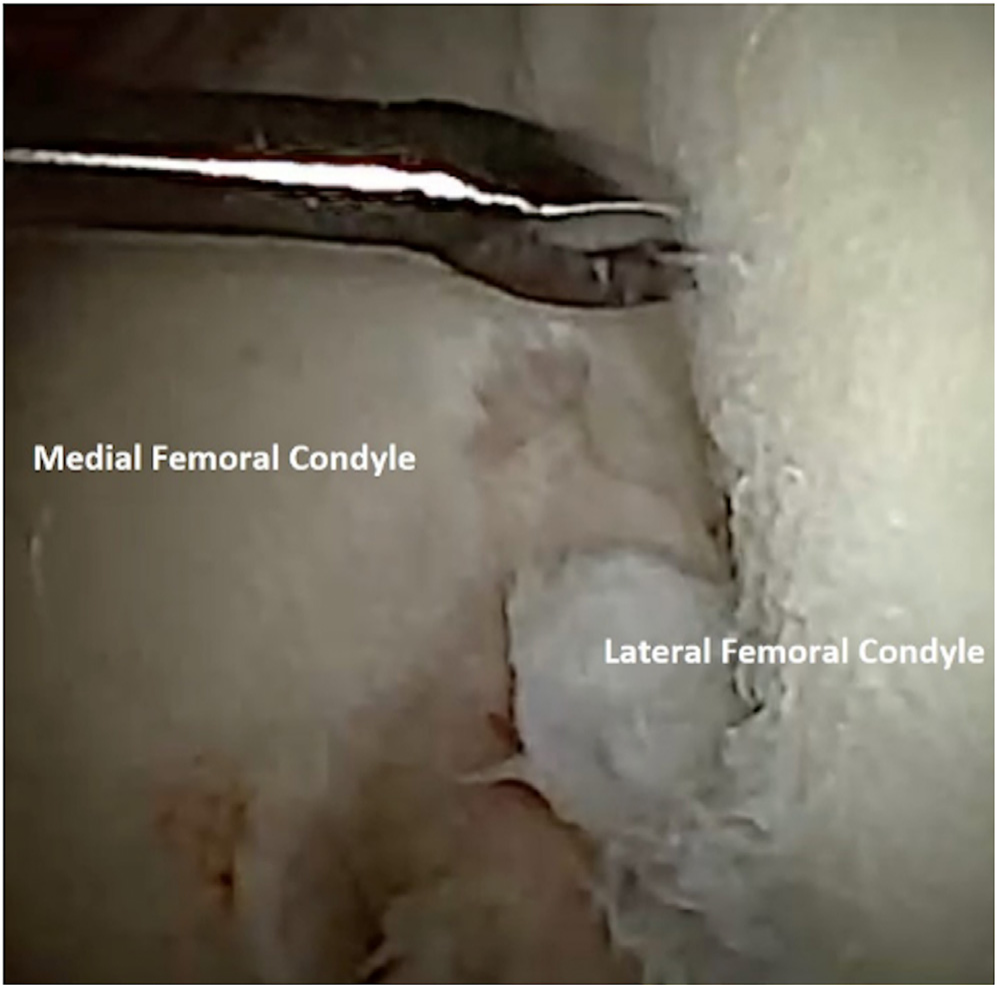
Needle arthroscopic view of left knee from anterolateral portal. The intercondylar notch can be visualized (middle). An area of stable cartilage in a non–weight-bearing region on the lateral femoral condyle (right) is identified for harvesting.

**Fig 4:**
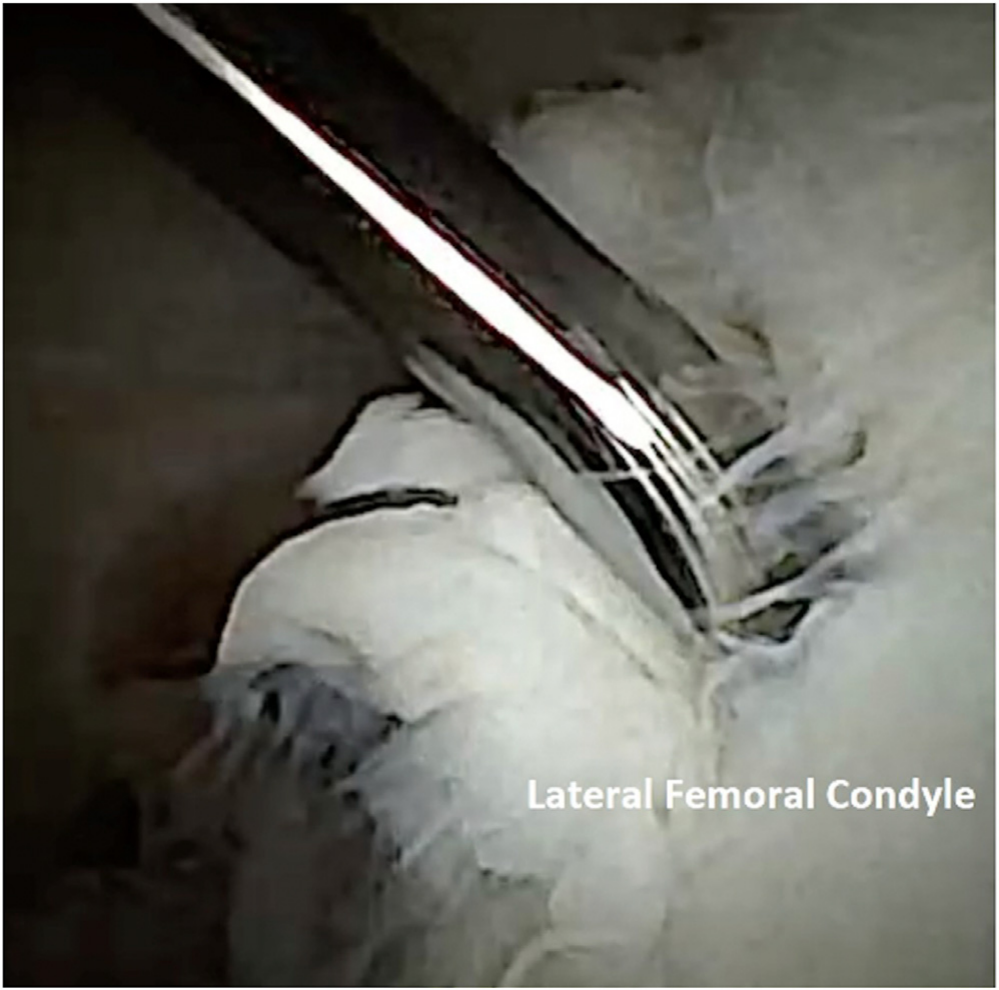
Needle arthroscopic intra-articular view of left knee from anterolateral portal. By use of a 2-mm Nano-elevator, the cartilage sample is obtained.

**Fig 5:**
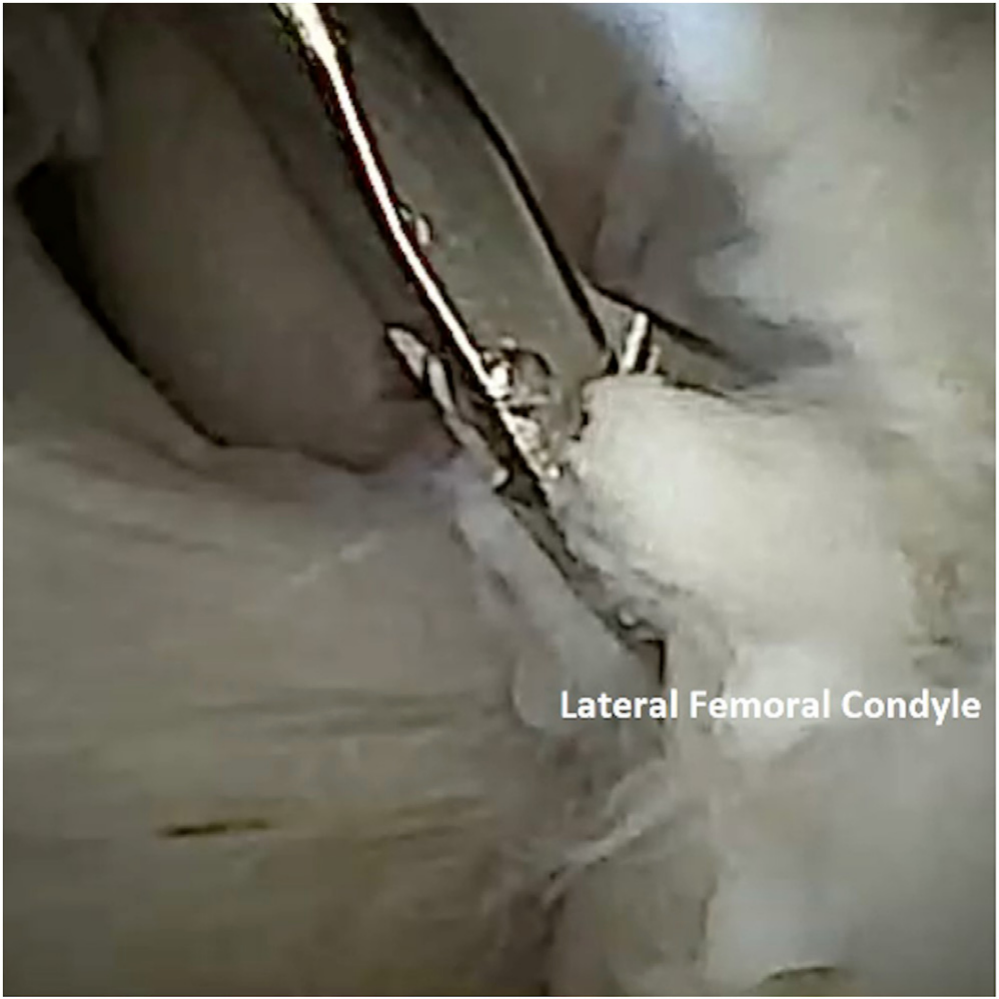
Needle arthroscopic intra-articular view of left knee from anterolateral portal. A 2-mm grasper is used to retrieve the cartilage specimen from the anteromedial portal (right).

**Fig 6:**
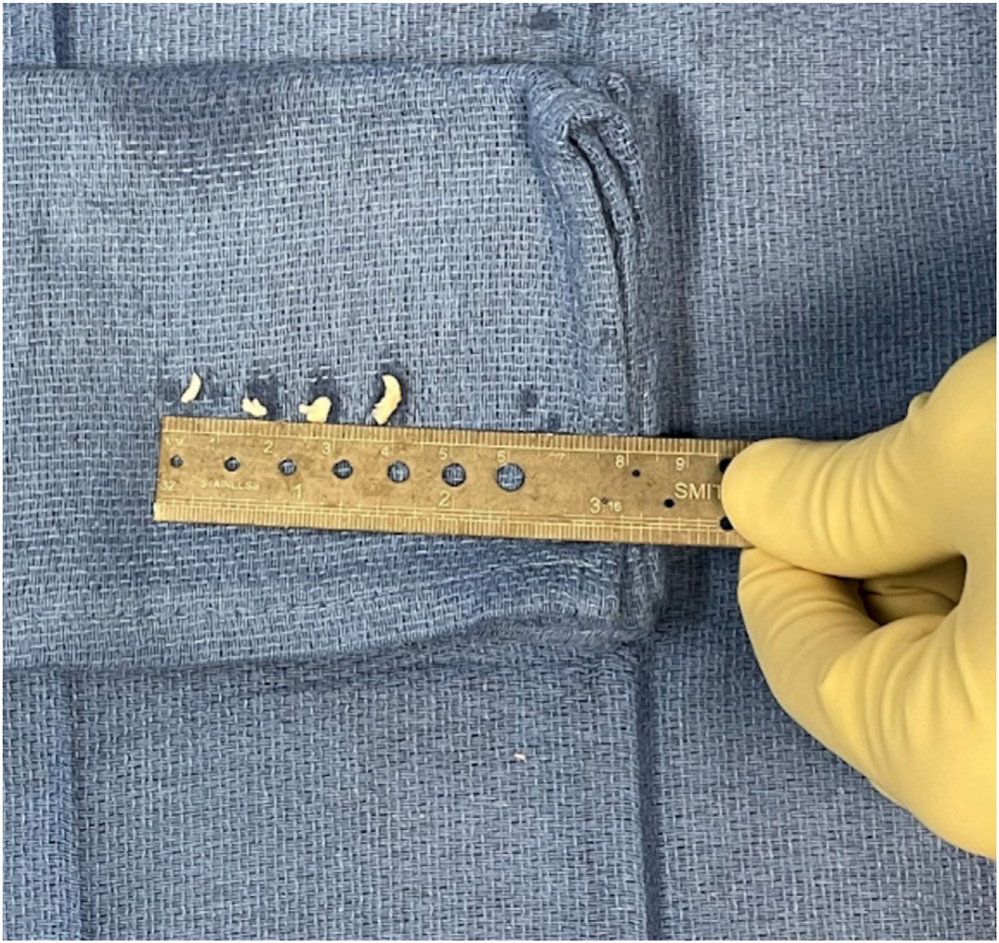
The obtained cartilage specimens are measured and subsequently placed in appropriate sterile medium for transport.

To perform chondrocyte harvest, attention is turned to the intercondylar notch (Fig 3). An 18-gauge spinal needle can be inserted back into the knee joint to allow for outflow of fluid, prioritizing the comfort of the awake patient. At the surgeon’s discretion, the optimal location for harvest is probed to assess the quality of the cartilage. A 2-mm Nano-elevator (Arthrex) is then used to lift the cartilage off the bone. It is important to note that full-thickness cartilage specimens should be obtained (Fig 4). A 2-mm grasper can be used to retrieve the cartilage specimen, which is then placed into sterile transport medium (Figs 5 and 6). Approximately 200 to 300 mg of healthy tissue is required for an adequate harvest.

The portals can be sealed primarily using adhesive wound closure strips (Steri-Strips; 3M, St Paul, MN) or with simple sutures at the surgeon’s discretion. A dry, sterile dressing is applied that facilitates early knee range of motion.

### Postoperative Protocol

The patient is allowed to fully bear weight and mobilize as tolerated and should be encouraged to perform range-of-motion exercises as instructed. The patient can also apply ice and elevate the knee when not ambulating for the first 24 to 72 hours to minimize swelling. No deep vein thrombosis prophylaxis or postoperative antibiotics are required. Acetaminophen and anti-inflammatories are sufficient for postoperative pain control.

## Discussion

The ability to use needle arthroscopy to perform wide-awake procedures provides a versatile tool for orthopaedic surgeons treating chondral pathologies. The interactive nature of the procedure allows increased patient involvement, education, and overall satisfaction with the procedure (Table 1). Additionally, the small size of the instruments leads to less trauma to the soft tissue and a potentially faster recovery. In this technical note, we describe the process of chondrocyte harvest for autologous cell–based repair of chondral lesions in the wide-awake setting. Although the video demonstration was performed in an operating room, the patient was wide awake with no regional or general anesthesia. Our goal is to provide a proof of concept that this technique can be replicated in the office setting (Tables 2 and 3).

**Table 1:** Advantages and Disadvantages of Technique

Advantages
Avoidance of potential risks associated with anesthesia
Ability to obtain cartilage biopsy specimen without 2 trips to operating room
Opportunity for improved patient-physician education and shared decision making
Reduced operating room resource utilization
Disadvantages
Potential for patient pain or discomfort
Learning curve associated 0° arthroscope
Unfamiliarity with operating on wide-awake patient

**Table 2:** Pearls and Pitfalls of Technique

Pearls
Perform in dedicated procedure room
Ensure familiarity of office staff with room setup, instrument turnover, and workflow
Perform thorough pre-procedure discussion with patient to assess mental readiness and expectations for wide-awake procedure
Allow at least 10 min between local anesthetic injection and initiation of procedure
Use epinephrine in saline solution to optimize hemostasis and visualization
Maintain communication with patient throughout procedure
Pitfalls
Not having all available assistants, instruments, and implants ready at beginning of procedure
Inappropriate patient selection for wide-awake procedure
Inadequate time between local anesthetic injection and initiation of procedure
Inappropriate portal placement for use with 0° needle arthroscope

**Table 3:** Step-by-Step Guide to Performing Technique

Step	Description
1	Position the patient comfortably in the supine position with the operative knee flexed slightly. Mark relevant surface anatomy and anticipated portals.
2	Deliver the local anesthetic cocktail into the sites of the anteromedial and anterolateral portals. Wait 10 min to ensure proper analgesia and minimize patient discomfort.
3	Establish the anterolateral portal with a stab incision. Avoid blunt dissection of the portal site.
4	Perform diagnostic arthroscopy of the knee. Maintain communication with the patient and discuss the observed pathology in real time.
5	Establish the anteromedial portal under direct visualization. Again, avoid blunt dissection of the portal site.
6	Ensure that the portals allow adequate access to the location of harvest (intercondylar notch). Note that an 18-gauge spinal needle can be inserted for outflow.
7	Use a 2-mm Nano-elevator to obtain a full-thickness cartilage specimen. Bear in mind that approximately 200-300 mg of tissue is required.
8	Use a 2-mm grasper to retrieve the cartilage specimen. Place the specimen into appropriate sterile transport medium.
9	Close the wounds using Steri-Strips or sutures, and apply a soft dressing.

With the advances in needle arthroscopy technology, a multitude of techniques have been reported recently. Shubert et al.^6^ described posterior cruciate ligament reconstruction using needle arthroscopy, which eliminated the need for 30° and 70° scopes. They reported infrequent reorientation and switching of portals and scopes, which increased operating room efficacy and decreased morbidity to the patient.^6^ Lavender et al.^7^ described single-incision rotator cuff repair using needle arthroscopy. Needle arthroscopy has also been described in the wide-awake office setting for diagnostic knee arthroscopy using both 1- and 2-portal techniques, as well as numerous foot and ankle procedures.^8^, ^9^, ^10^, ^11^, ^12^, ^13^, ^14^, ^15^ In a cohort of 31 patients undergoing in-office needle arthroscopy debridement for anterior ankle impingement, Colasanti et al.^16^ reported significant improvement in patient-reported outcome scores, a low complication rate, and faster recovery compared with traditional arthroscopic debridement. They also reported high patient satisfaction with the procedure, with patients reporting a willingness to undergo the same procedure again.^16^

The increasing utilization and description of needle arthroscopy in the literature have illuminated its potential. As shown, an area that would likely benefit from this technology is cell-based articular cartilage restoration procedures. Although chondral lesions of the knee pose a challenging pathology to treat, recent advancements in autologous cell–based cartilage repair techniques have shown promising results. Meyerkort et al.^17^ retrospectively reviewed a cohort of 23 patients with a mean age of 42 years who underwent MACI for chondral defects of the patellofemoral joint. At 5 years postoperatively, there was a significant improvement in functional outcomes and 91% of patients reported a willingness to undergo the procedure again.^17^ Colombini et al.,^4^ in their systematic review of both autologous chondrocyte implantation (ACI) and MACI, showed stable clinical improvements at 11 to 15 years postoperatively, with a failure rate of only 10% at 11 years.

Despite the promising medium- and long-term results of these cell-based therapies, some critics have called into question the cost and additional utilization of hospital resources of these procedures. In a cost analysis model, LeBrun et al.^18^ determined that MACI added 6.92 quality-adjusted life-years although at a cost of $83,073 owing to the 2-stage nature and expense of the graft. Vogelmann et al.,^5^ however, developed a discrete-event simulation of over 10,000 patients in the German health care system with an average chondral defect size of 4.5 cm^2^. They determined that over the average modeled time of 48 years, MACI was a highly cost-effective treatment, with an added 1.32 quality-adjusted life-years compared with cases treated without MACI.^5^

The use of in-office wide-awake needle arthroscopy for the first stage of cell-based cartilage repair therapies would substantially reduce cost, utilization of hospital resources, and exposure to anesthesia for the patient. In a retrospective review of 200 patients, McMillan et al.^19^ compared the cost of a diagnostic in-office needle arthroscopy versus noncontrast magnetic resonance imaging. They reported an average savings of $961.08 per patient for the knee and $1,097.62 per patient for the shoulder compared with magnetic resonance imaging performed in hospital-based facilities, further highlighting the potential of this technology.^19^

In conclusion, wide-awake needle arthroscopy is an effective diagnostic and therapeutic tool that provides another technique for orthopaedic surgeons to use when treating chondral pathologies. Specifically, the cartilage harvest aspect of a 2-stage ACI procedure can be safely performed in the wide-awake patient with only local anesthesia. In our experience, we found the ease of use and the ability to speak with the patient during the procedure advantageous. This technical note serves as a proof of concept to describe cartilage harvest for ACI in the wide-awake setting. Needle arthroscopy has the potential to reduce cost, decrease utilization of health care resources, and promote a faster recovery.

## Supplementary Data

ICMJE author disclosure forms

Video 1 Demonstrates cartilage biopsy for autologous cell-based repair of the knee in the wide awake setting using needle arthroscopy.
